# Prevalence, Factors, and Association of Electronic Communication Use With Patient-Perceived Quality of Care From the 2019 Health Information National Trends Survey 5-Cycle 3: Exploratory Study

**DOI:** 10.2196/27167

**Published:** 2022-02-04

**Authors:** Rumei Yang, Kai Zeng, Yun Jiang

**Affiliations:** 1 School of Nursing Nanjing Medical University Nanjing China; 2 School of Nursing Southern Medical University Guangzhou China; 3 School of Nursing University of Michigan Ann Arbor, MI United States

**Keywords:** electronic communication, quality of care, person-related characteristics, patient preference, HINTS

## Abstract

**Background:**

Electronic communication (e-communication), referring to communication through electronic platforms such as the web, patient portal, or mobile phone, has become increasingly important, as it extends traditional in-person communication with fewer limitations of timing and locations. However, little is known about the current status of patients’ use of e-communication with clinicians and whether the use is related to the better patient-perceived quality of care at the population level.

**Objective:**

The aim of this study was to explore the prevalence of and the factors associated with e-communication use and the association of e-communication use with patient-perceived quality of care by using the nationally representative sample of the 2019 Health Information National Trends Survey 5 (HINTS 5)-Cycle 3.

**Methods:**

Data from 5438 adult responders (mean age 49.04 years, range 18-98 years) were included in this analysis. Multiple logistic and linear regressions were conducted to explore responders’ personal characteristics related to their use of e-communication with clinicians in the past 12 months and how their use was related to perceived quality of care. Descriptive analyses for e-communication use according to age groups were also performed. All analyses considered the complex survey design using the jackknife replication method.

**Results:**

The overall prevalence of e-communication use was 60.3%, with a significantly lower prevalence in older adults (16.6%) than that in <45-year-old adults (41%) and 45-65-year-old adults (42.4%). All percentages are weighted; therefore, absolute values are not shown. American adults who used e-communication were more likely to be high school graduates (odds ratio [OR] 1.95, 95% CI 1.14-3.34; *P=*.02), some college degree holders (OR 3.34, 95% CI 1.84-6.05; *P*<.001), and college graduates or more (OR 4.89, 95% CI 2.67-8.95; *P*<.001). Further, people who were females (OR 1.47, 95% CI 1.18-1.82; *P*=.001), with a household income ≥US $50,000 (OR 1.63, 95% CI 1.23-2.16; *P=*.001), with more comorbidities (OR 1.22, 95% CI 1.07-1.40; *P*=.004), or having a regular health care provider (OR 2.62, 95% CI 1.98-3.47; *P*<.001), were more likely to use e-communication. In contrast, those who resided in rural areas (OR 0.61, 95% CI 0.43-0.88; *P=*.009) were less likely to use e-communication. After controlling for the sociodemographics, the number of comorbidities, and relationship factors (ie, having a regular provider and trusting a doctor), e-communication use was found to be significantly associated with better perceived quality of care (β=.12, 95% CI 0.02-0.22; *P*=.02).

**Conclusions:**

This study confirmed the positive association between e-communication use and patient-perceived quality of care and suggested that policy-level attention should be raised to engage the socially disadvantaged (ie, those with lower levels of education and income, without a regular health care provider, and living in rural areas) to maximize e-communication use and to support better patient-perceived quality of care among American adults.

## Introduction

Effective patient-clinician communication is a critical component of high-quality patient-centered care. With the rapid diffusion of advanced technology, the use of electronic services such as email, text messaging, and patient portals as a platform of communication (ie, electronic communication [e-communication]) between patients and clinicians has become increasingly popular [1]. Evidence shows that patients are enthusiastic about e-communication with clinicians regarding a wide variety of clinical contexts such as chronic condition self-management and follow-up examinations [2-4]. e-Communication has become a valuable supplement to traditional in-person communication through office visits [5,6]. It has fundamentally improved patients’ interactions with the health care system and their engagement in shared decision-making with clinicians [7,8].

Despite the increasing popularity and potential impacts of e-communication on health care services, the actual use of e-communication among various patient populations still remains relatively low [9-12]. A review of patient portals for adults with diabetes found that 29%-46% of adults registered an account, but only 27%-76% of them actually accessed the portal [12]. A study of an encrypted message system in a pediatric clinic showed that only 4.3% of parents of chronically ill children made use of the system [11]. Similarly, a study of Health Information National Trends Survey 5 (HINTS 5)-Cycle 3 data in 2003-2005 indicated that only 10% of adult internet users communicated with the clinicians through web-based communication services [9]. Age can be a potential factor affecting the use of e-communication [13,14]. Clarke et al’s [14] study showed that young adults preferred text messaging, middle-aged adults preferred phone calls, and older adults preferred paper-based and in-person interactions with clinicians. These findings imply that the prevalence of e-communication use might be lower among older adults as compared to that among young and middle-aged adults. Considering older adults’ needs for technology-enabled health care support can help them become the major users of e-communication. In recent years, older adults’ adoption of information and communication technology has been increasing, and they are likely to increasingly incorporate digital technology into their daily life [15]. Given the ever evolving technology and various populations’ needs for support, it is important to understand how e-communication use varies across different age groups. Another commonly reported factor associated with patients’ use of e-communication is patient-clinician relationships [16-20], for example, how much one trusts information from a doctor can influence the person’s decision-making for using e-communication [19].

All these barriers can presumably affect both patients’ use of e-communication [20] and their perceptions of quality of care [21]. However, there lacks empirical evidence to quantify the association between e-communication use and patient-perceived quality of care [22,23]. Patient-perceived quality of care refers to patients’ perception of health care services received based on their experiences of what actually happened during the care process [24]. As one of the essential indicators of care quality, patients’ perception of quality of care is an important driver of patient satisfaction, reflecting their desire for individualized high-quality care, which is also the main goal for those providing the care [25]. Factors that affect patient-perceived quality of care mainly include person-related conditions such as the patients’ age, sex, education level, and self-reported health status, and external objective care conditions such as the organizational structure of care, competence of health care personnel, the size of the hospital, inpatient stay and occupancy, and comfortable environment [26]. Patient-clinician communication has been reported as one of the major factors driving patient perception of quality care in addition to hospital staff responsiveness, the care transition process, and hospital environment [27]. In the era of digital health, particularly with the increased popularity of e-communication between patients and clinicians and extended health care efficiency, the use of e-communication may increase the patient-perceived quality of care as opposed to no use of any e-communication [21]. However, the lack of empirical evidence to quantify the effectiveness of e-communication on patient-perceived quality of care may delay the promotion of e-communication adoption and the development of new models of patient-clinician interaction to satisfy patients’ needs for high-quality health care services [21].

The purposes of this study were to examine the prevalence of patients’ use of e-communication with clinicians and the potential factors (in particular, person-related factors such as age) associated with their use of e-communication and to explore the potential association between e-communication use and patient-perceived quality of care. Based on previous literature reports [21,24,25,27], we hypothesized that patients’ use of e-communication was related to better patient-perceived quality of care.

## Methods

### Data Source

Data used in this study were from the HINTS 5-Cycle 3 [28]. HINTS is a nationally representative survey designed to understand American adults’ knowledge of, attitudes toward, and use of cancer- and health-related information [29]. HINTS 5-Cycle 3 used a single-mode mail survey, with a 2-stage sample design, including a stratified sample of addresses and a selected adult within each sampled household [28]. The data were collected from 5438 respondents from January to May 2019 (English version only), with an overall 30.3% response rate [28]. Comprehensive reports on the sampling design for the HINTS survey have been published elsewhere [28-30]. The survey data were deidentified and are publicly available; institutional review board approval was not applicable.

### Variables

#### Perceived Quality of Care

The outcome variable patient-perceived quality of care was assessed via self-report on a single question asking “*overall, how would you rate the quality of health care you received in the past 12 months?*” with a 5-point Likert scale from 1=poor to 5=excellent, with a high score indicating better perceived quality of care (see Table 1).

**Table 1:** Variables and survey measurements.

Variable			Survey measurement
Patient-perceived quality of care			Overall, how would you rate the quality of health care you received in the past 12 months? (1=poor to 5=excellent)
**Use of electronic communication**			
	1	In the past 12 months, have you used a computer, smartphone, or other electronic means to communicate with a doctor or a doctor’s office? (1=yes, 0=no)	
	2	Have you sent a text message to or received a text message from a doctor or other health care professional within the last 12 months? (1=yes, 0=no)	
	3	In the past 12 months, have you used your online medical record to securely message health care provider and staff (eg, email)? (1=yes, 0=no)	
	4	In the past 12 months, have you used your online medical record to add health information to share with your health care provider, such as health concerns, symptoms, and side effects? (1=yes, 0=no)	
	5	Have you shared health information from either an electronic monitoring device or smartphone with a health professional within the last 12 months? (1=yes, 0=no)	
	6	Have you electronically sent your medical information to another health care clinician? (1=yes, 0=no).	
**Sociodemographics**			
	1	Age (young: ≥18 and <45 years, middle-aged: ≥45 and <65 years, and older adults ≥65 years)	
	2	Sex (0=male, 1=female)	
	3	Education level (0=less than high school, 1=high school graduate, 2=some college, 3=college graduate or more)	
	4	Marital status (0=not married, 1=married or partnered)	
	5	Race/ethnicity (0=White, 1=African American, 2=Hispanic, 3=other)	
	6	Household income (0=<US $50,000; 1=≥US $50,000)	
	7	Living status (0=living with others, 1=living alone)	
	8	Residency (0=nonrural, 1=rural)	
Comorbidities			The number of comorbidities: Has a doctor or other health professional ever told you that you had any of the following medical conditions? Choices for this question included cancer, hypertension, diabetes, heart condition, chronic lung disease, and depression, and a sum score was used.
**Patient-clinician relationship**			
	1	Having a regular health care provider: Not including psychiatrists and other mental health professionals, is there a particular doctor, nurse, or other health professional that you see most often? (0=no, 1=yes)	
	2	Trusting a doctor: In general, how much would you trust information about health or medical topics from a doctor? (1=not at all to 4=a lot)	

#### Use of e-Communication

Patients’ use of e-communication with clinicians in the past 12 months, such as using the computer, smartphone, text messaging, web-based messaging, web-based medical records, or any other electronic means to share medical information, were assessed through 6 survey questions (see Table 1). Survey responders who answered “yes” to either of the 6 questions were considered having e-communication with their clinicians, defined as users, while responders who answered “no” to all 6 questions were considered as nonusers.

#### Sociodemographics and Comorbidities

Age was measured as a continuous variable in the HINTS 5-Cycle 3 and was categorized into 3 groups: young adults (≥18 and <45 years of age, 38.4%), middle-aged adults (≥45 and <65 years of age, 39.7%), and older adults (≥65 years of age, 19.7%). All percentages are weighted; therefore, absolute values are not shown. Other sociodemographic covariates included sex, education level, marital status, race/ethnicity, household income, living status, and residency. The number of comorbidities was a sum score of 6 doctor-diagnosed chronic conditions, namely, cancer, diabetes, hypertension, heart disease, lung disease, and depression (see Table 1).

#### Patient-Clinician Relationship

Patient-clinician relationship variables included (1) having a regular health care provider (yes/no) and (2) trusting a doctor (rating from 1=not at all to 4=a lot) (see Table 1).

### Statistical Analysis

All analyses considered the complex survey design of the HINTS 5-Cycle 3 sample by using the HINTS-supplied final weights to estimate population estimates and 50 replicate weights to compute the standard errors with the jackknife replication approach [29]. Specifically, descriptive statistics were used to describe the prevalence and the characteristics of e-communication users and nonusers. Multiple logistic regression analyses were used to assess the association of sociodemographics and comorbidities (Model 1) and sociodemographics, comorbidities, plus patient-clinician relationship factors (Model 2) with e-communication use. Multiple linear regression analyses were used to examine the association between e-communication use and patient-perceived quality of care with the control of sociodemographics and comorbidities (Model 3) and the control of sociodemographics, comorbidities, plus patient-clinician relationship factors (Model 4). Missing data pattern analysis indicated that most variables had missing data <5% (see Table S1 in Multimedia Appendix 1). Multiple imputation was performed, and the pooled results of model 3 and model 4 were based on 50 imputed data sets using multiple imputation by chained equations. All analyses were conducted using Stata software (version 14; StataCorp). Results were reported as weighted point estimates and 95% CIs. The level of significance was .05.

## Results

### Prevalence and Characteristics of e-Communication Users

The overall prevalence of the use of e-communication was 60.3%. Most American adults who used e-communication with clinicians in the past 12 months were younger than 65 years, as older adults only accounted for 16.6% of e-communication users but 25.7% of nonusers (see Table 2). Table 2 also displays that most e-communication users were females (53.9%), had at least some college (41.7%), and 36.4% college graduates or more, were White people (65%), currently married (59.9%), with a household income ≥US $50,000 (63.9%), and did not live alone (85%) or in rural areas (89.5%). e-Communication users and nonusers were significantly different in all person-related characteristics. In addition, significantly more e-communication users had a regular health care provider than e-communication nonusers (72.9% vs 51.4%, respectively; *P*<.001) (see Table 2). All percentages are weighted; therefore, absolute values are not shown.

**Table 2:** Sociodemographic characteristics and comorbidities of electronic communication users versus nonusers.^a^

Characteristics			All users (N=5438)		Nonusers (n=2092)		Users (n=3337)		*P* value
Age (years), mean (SD)			49.58 (17.58)		50.52 (19.06)		48.06 (16.36)		*.005*
Comorbidities, mean (SD)			1.12 (1.15)		0.99 (1.14)		1.08 (1.13)		.14
Trusting a doctor, mean (SD)			3.67 (0.58)		3.56 (0.66)		3.66 (0.59)		*.001*
Patient-perceived quality of care, mean (SD)			3.96 (0.93)		3.84 (0.92)		4.01 (0.93)		*.002*
**Age categories (% weighted)^b^**									*<.001*
	Young adults (<45 years)	38.4		36.5		41.0			
	Middle-aged adults (45-64 years)	39.7		37.9		42.4			
	Older adults (≥65 years)	19.7		25.7		16.6			
Gender (female) (% weighted)^c^			50.1		47.2		53.9		*.003*
**Education level (% weighted)^d^**									*<.001*
	Less than high school	6.8		12.2		3.6			
	High school graduate	22.8		31.3		18.3			
	Some college	39.1		37.7		41.7			
	College graduate or more	28.7		18.7		36.4			
Marital status (married or partnered, % weighted)^e^			54		49.1		59.9		*<.001*
**Race/ethnicity (% weighted)^f^**									*.002*
	White	58		60.9		65			
	African American	10.3		13.1		10.2			
	Hispanic	15.4		20		14.9			
	Other	7.7		6		9.9			
Household income (≥US $50,000) (% weighted)^g^			54.5		42.2		63.9		*<.001*
Living alone (% weighted)^h^			16.9		21.9		15		*<.001*
Residency (rural) (% weighted)^i^			13.3		17.4		10.5		*.001*
Having a regular health care provider (yes) (% weighted)^j^			63.3		51.4		72.9		*<.001*
Use of electronic communication (yes) (% weighted)^k^			60.3		—^l^		—		—

^a^Absolute values are not provided in this table because the percentages are weighted. The absolute values are summarized in the Multimedia Appendix 2. Significant *P* values are italicized.

^b^Age categories (0=young adults, 1=middle-aged adults, 2=older adults).

^c^Gender (0=male, 1=female).

^d^Education (0=less than high school, 1=high school graduate, 2=some college, 3=college graduate or more).

^e^Marital status (0=not married, 1=married or partnered).

^f^Race/ethnicity (0= White, 1=African American, 2=Hispanic, 3=other).

^g^Household income (0=less than US $50,000, 1=≥US $50,000).

^h^Living alone (0=living with others, 1=living alone).

^i^Residency (0=nonrural, 1=rural).

^j^Having a regular health care provider (0=no, 1=yes).

^k^Use of electronic communication with a clinician (0=no, 1=yes).

^l^Not available.

### Factors Associated With e-Communication

Table 3 presents the results of multiple logistic regression analyses on the sociodemographics, comorbidities, and patient-clinician relationship factors for e-communication use. In model 1, where only sociodemographic factors and comorbidities were considered, age (odds ratio [OR] 0.87, 95% CI 0.66-1.14), female (OR 1.44, 95% CI 1.17-1.77), education level (eg, for college graduates or more, OR 4.78, 95% CI 2.63-8.68), household income (OR 1.77, 95% CI 1.34-2.34), rural residency (OR 0.62, 95% CI 0.44-0.87), and number of comorbidities (OR 1.33, 95% CI 1.16-1.52) were associated with e-communication use (see Table 3). In model 2, after adding the relationship factors to the model, people who were females (OR 1.47, 95% CI 1.18-1.82), high school graduates (OR 1.95, 95% CI 1.14-3.34), having some college (OR 3.34, 95% CI 1.84-6.05), and college graduates or more (OR 4.89, 95% CI 2.67-8.95), with a household income at or greater than US $50,000 (OR 1.63, 95% CI 1.23-2.16), with more comorbidities (OR 1.22, 95% CI 1.07-1.40), or having a regular health care provider (OR 2.62, 95% CI 1.98-3.47) were more likely to use e-communication, whereas those who were older adults (OR 0.42, 95% CI 0.31-0.57) or rural residents (OR 0.61, 95% CI 0.43-0.88) were less likely to use e-communication.

**Table 3:** Factors associated with electronic communication.

Variables			Model 1^a^					Model 2^b^		
			Odds ratio (95% CI)		*P* value^c^		Odds ratio (95% CI)			*P* value^c^
**Age**										
	Young adults (<45 years)	Ref^d^		Ref		Ref			Ref	
	Middle-aged adults (45-64 years)	0.87 (0.66-1.14)		.31		0.86 (0.65-1.15)			.30	
	Older adults (≥65 years)	0.51 (0.39-0.68)		*<.001*		0.42 (0.31-0.57)			*<.001*	
Female			1.44 (1.17-1.77)		*.001*		1.47 (1.18-1.82)			*.001*
**Education level**										
	Less than high school	Ref		Ref		Ref			Ref	
	High school graduate	1.92 (1.09-3.39)		*.03*		1.95 (1.14-3.34)			*.02*	
	Some college	3.32 (1.82-6.07)		*<.001*		3.34 (1.84-6.05)			*<.001*	
	College graduate or more	4.78 (2.63-8.68)		*<.001*		4.89 (2.67-8.95)			*<.001*	
Married or partnered			1.28 (0.97-1.69)		.08		1.26 (0.94-1.68)			.12
**Race/ethnicity**										
	White	Ref		Ref		Ref			Ref	
	African American	0.93 (0.62-1.38)		.70		1.03 (0.68-1.57)			.89	
	Hispanic	0.86 (0.63-1.17)		.32		1.04 (0.76-1.41)			.81	
	Other	1.44 (0.93-2.21)		.10		1.55 (1.01-2.39)			.05	
Household income (≥US $50,000)			1.77 (1.34-2.34)		*<.001*		1.63 (1.23-2.16)			*.001*
Living alone			0.94 (0.69-1.29)		.70		0.95 (0.68-1.31)			.74
Rural residency			0.62 (0.44-0.87)		*.008*		0.61 (0.43-0.88)			*.009*
Number of comorbidities			1.33 (1.16-1.52)		*<.001*		1.22 (1.07-1.40)			*.004*
Having a regular health care provider (yes)			—^e^		—		2.62 (1.98-3.47)			*<.001*
Trusting a doctor			—		—		1.14 (0.96-1.37)			.14

^a^Model 1 adjusted for sociodemographic factors (eg, age categories, gender, education, marital status, race/ethnicity) and comorbidities.

^b^Model 2 adjusted for sociodemographics, comorbidities, plus relationship factors (eg, having a regular health care provider, trust a doctor).

^c^Significant *P* values are italicized.

^d^Ref: reference value.

^e^Not available.

### Associations Between e-Communication Use and Patient-Perceived Quality of Care

Table 4 displays the results of the association between e-communication use and patient-perceived quality of care among American adults. After controlling for sociodemographic factors (age, gender, education, income), comorbidities, and patient-clinician relationship factors (having a regular health care provider, trust a doctor), the use of e-communication was statistically associated with better quality of care (β=.12, 95% CI 0.02-0.22; see Model 4 in Table 4).

**Table 4:** Association between electronic communication and patient-perceived quality of care based on 50 imputed data sets using chained equations.

Variables		Model 3^a^				Model 4^b^		
		β	95% CI	*P* value^c^	β		95% CI	*P* value^c^
Use of electronic communication		.20	0.09 to 0.30	*<.001*	.12		0.02 to 0.22	*.02*
**Age^d^**								
	Young adults	Ref^e^	Ref	Ref	Ref		Ref	Ref
	Middle-aged adults	.06	–0.06 to 0.17	.36	.06		–0.05 to 0.17	.27
	Older adults	.24	0.12 to 0.36	*<.001*	.17		0.05 to 0.28	*.005*
Female		.00	–0.10 to 0.09	.92	–.01		–0.10 to 0.08	.84
**Education level**								
	Less than high school	Ref	Ref	Ref	Ref		Ref	Ref
	High school graduate	.09	–0.30 to 0.12	.39	–.07		–0.26 to 0.12	.45
	Some college	–.07	–0.27 to 0.14	.51	–.09		–0.28 to 0.10	.32
	College graduate or more	.01	–0.20 to 0.22	.90	–.05		–0.24 to 0.14	.62
	Married or partnered	.03	–0.11 to 0.17	.65	.04		–0.09 to 0.17	.53
**Race/ethnicity**								
	White	Ref	Ref	Ref	Ref		Ref	Ref
	African American	–.07	–0.24 to 0.09	.39	–.01		–0.17 to 0.14	.86
	Hispanic	.00	–0.15 to 0.15	.96	.06		–0.08 to 0.20	.40
	Other	–.25	–0.46 to –0.04	*.02*	–.23		–0.44 to –0.03	*.02*
Household income (≥US $50,000)		.11	–0.01 to 0.23	.08	.08		–0.04 to 0.19	.19
Living alone		.05	–0.11 to 0.21	.55	.07		–0.08 to 0.22	.38
Rural residency		–.02	–0.18 to 0.15	.82	–.03		–0.20 to 0.13	.70
Number of comorbidities		–.06	–0.11 to –0.00	*.03*	–.08		–0.12 to –0.03	*.001*
Having a regular health care provider		—^f^	—	—	.47		0.39 to 0.55	*<.001*
Trusting a doctor		—	—	—	.20		0.10 to 0.31	*<.001*

^a^Model 3 adjusted for sociodemographic factors (eg, age categories, gender, education, marital status, race/ethnicity) and comorbidities.

^b^Model 4 adjusted for sociodemographics, comorbidities, plus relationship factors (eg, having a regular health care provider, trusting a doctor).

^c^Significant *P* values are italicized.

^d^Age categories: young adults (<45 years), middle-aged adults (45-64 years), older adults (≥65 years).

^e^Ref: reference value.

^f^Not available.

## Discussion

### Principal Findings

This study examined the prevalence of and factors associated with e-communication use and the potential association between e-communication use and patient-perceived quality of care in a nationally representative sample of American adults. To the best of our knowledge, this study is the first to explore the association of e-communication use with patient-perceived quality of care at the population level. Several important findings emerged in this study.

First, the majority of American adults (60.3%) used some forms of e-communication with clinicians throughout 2019, which was significantly higher than the reported 7% in 2003, 10% in 2005 [9], and 31.5% in 2014 from the previous HINTS [31]. This finding indicates that e-communication use has become increasingly popular for adults to interact with their clinicians. The increased prevalence rate can be attributed to the increased availability and popularity of electronic health devices [32,33] and supportive policies (eg, promoting patient access to their electronic medical records) [34]. Although our data showed an overall growing trend in the use of e-communication, it is important to note that older adults’ use of e-communication still remained relatively low, and this rate was not much improved from that in 2003 and 2005 [12]. Literature indicates that older adults usually prefer direct in-person interactions with their clinicians [12], while there are increasing reports about older adults’ positive attitude toward e-communication and their preference for email and messaging communication with clinicians that is similar to that for younger adults [35,36]. Our finding suggests that there is still a gap in the actual use of e-communication between older adults and young adults [31,37,38]. More studies are needed to explore the practical challenges that older adults may encounter in the use of e-communication. Older adults are potentially the major users of e-communication, considering their high level of health care needs. It is important to develop appropriate e-communication support for this population for their better health outcomes.

In addition to age, we also found that the use of e-communication varied by gender, education, income, and residency, indicating that individuals who are females, with higher education, higher income, and more comorbidities, or who reside in nonrural areas were more like to use e-communication with their clinicians. This finding is congruent with reports of the general adoption of eHealth in literature [39-42]. Consistent with our finding, the positive association between education and e-communication usage was reported in previous studies [31,39], which can be interpreted as individuals who have higher education might have more eHealth literacy skills and technological capabilities [43] to help them better use electronic forms of information [31,39]. However, Senft and Everson’s recent study [44] reported that individuals who had lower levels of education and had negative care coordination experiences are more likely to use eHealth activities to communicate with clinicians [44], indicating that personal health care experiences can possibly interplay with education and thus influence the use of e-communication. However, it is unclear whether the limited use of e-communication among rural residents is related to lack of internet connectivity or awareness of e-communication services [45]. Additional studies can be conducted for further exploration.

Compared to those who did not use e-communication in the past year, in this study, e-communication users were more likely to have a regular health care provider and reported better trust in information from a doctor. However, trusting a doctor was not an independent predictor of e-communication use when having a regular health care provider was controlled for in the model. A previous qualitative study has indicated that a trusting relationship between patient-clinician is a significant contributor to better online patient-clinician interactions [20,46]. Even those who tend to frequently seek web-based health information are more willing to use the information provided by their trusted clinicians for their health decision-making [17,47]. Our findings suggested that patients with a regular health care provider had the greatest association with their use of e-communication. It is possible that patients who have a regular health care provider have already built a trusting relationship with their clinicians. Given the importance of trust in a provider in the patient-centered care process, future research directly examining possible confounding of this factor using longitudinal data is recommended.

Finally, it is not surprising that this study found that the use of e-communication was an independent predictor of patient-perceived quality of care. In 2001, the Institute of Medicine suggested that e-communication could improve the quality of care [48]. The previous literature review demonstrates that e-communication provides a convenient way of patient-clinician interaction, has a positive impact on patient satisfaction while saving time for patients and clinicians, and has the potential to extend health care efficiency [21,49]. The benefits and challenges of e-communication have been well addressed in the literature, while its benefits for the quality of care may not have been clearly quantified previously. The measures of quality of care can vary by the dimensions of care and care processes [50]. However, this study focused on the measure of the patient-perceived quality of care, which solely reflected patients’ perceptions of health care services received based on their experiences of care [24]. It did not mean to measure any technical clinical quality, for example, cholesterol screening [51]. There is increasing interest in patient-reported measures, as experiences with care are more easily understood by patients. In addition, previous literature demonstrated that the measure of patient experiences of care was related to measures of the technical quality of care, which can serve as valid summary measures of hospital quality [52]. These study findings were based on the analysis of nationally representative survey data, which should be generalizable to all American adults. The positive association between the use of e-communication and perceived quality of care confirms that e-communication can serve as an important tool to improve patient satisfaction and their perceptions of quality of care. This finding is particularly significant and applicable in the current COVID-19 pandemic when traditional in-person communication is less feasible. It is expected that e-communication will continuously replace an adequate portion of traditional face-to-face encounters and has the potential to transform the health care system [21]. Future research can be conducted to explore the sustainable long-term effects of e-communication on patient-centered care outcomes.

### Limitations

This study has a few limitations. First, data were mainly based on self-reports, which might have introduced recall bias. Second, the survey questions regarding the use of e-communication did not specify the frequency of use; therefore, they did not accurately reflect responders’ experiences of using e-communication and might affect their perceptions of quality of care. Third, a binary measure of e-communication use (yes/no) was used, which might result in the loss of information or power. However, considering the conceptual overlaps across 6 questions about e-communication behaviors in the survey, a combined continuous assessment for the number of e-communication behaviors would be conceptually inaccurate. Fourth, the e-communication was between patients and clinicians. However, the survey only focused on the patient side and thus, it was not possible to know clinicians’ perceptions of e-communication use. Finally, the results could be underestimated by potential reverse causality owing to the nature of the study design. The prevalence of e-communication use was higher in our study than that that reported in previous studies. The difference may also be due to varying measurement methods across studies. In our study, we used 6 questions to measure e-communication, which are more than that used in other studies. Different measures might affect comparisons of the prevalence of e-communication use across studies.

### Conclusions

American adults’ use of e-communication with clinicians has been significantly increased in the past decade, which may be due to increased patient needs and advanced support from technologies and policies. As a convenient way of patient-clinician interaction, the use of e-communication is significantly associated with patient-perceived quality of care. The findings of multiple factors associated with e-communication use and the positive association between e-communication use and patient-perceived quality of care suggest that policy-level attention is needed to engage the socially disadvantaged (ie, those with lower levels of education and income, without a regular health care provider, and living in rural areas) to maximize the use of e-communication and to support better patient-perceived quality of care among American adults.

## Appendix

Multimedia Appendix 1 Missing data information.

Multimedia Appendix 2 Absolute value data for weighted percentages.

## Abbreviations

e-Communication: electronic communication
HINTS: Health Information National Trends Survey
OR: odds ratio
