# Supplementary material for: Prevalence, Factors, and Association of Electronic Communication Use With Patient-Perceived Quality of Care From the 2019 Health Information National Trends Survey 5-Cycle 3: Exploratory Study
Source: J Med Internet Res. 2022 Feb 4;24(2):e27167. doi: 10.2196/27167 (PMC8857700; doi:10.2196/27167)
Supplement: Multimedia Appendix 1 [file jmir_v24i2e27167_app1.docx]

# Table S1. Missing data information (N=5438)

|  | Missing, n (%) |
| --- | --- |
| Quality of care | 724 (13.3) |
| Electronic communication | 9 (0.2) |
| Age | 154 (2.8) |
| Gender | 129 (2.4) |
| Education | 157 (2.9) |
| Marital status | 171 (3.1) |
| Race/Ethnicity | 588 (10.8) |
| Household income | 73 (1.3) |
| Living alone | 264 (4.9) |
| Residency | 0 (0) |
| Comorbidity | 95 (1.7) |
| Had a regular provider | 105 (1.9) |
| Trust a provider | 143 (2.6) |
