# Supplementary material for: Prevalence, Factors, and Association of Electronic Communication Use With Patient-Perceived Quality of Care From the 2019 Health Information National Trends Survey 5-Cycle 3: Exploratory Study
Source: J Med Internet Res. 2022 Feb 4;24(2):e27167. doi: 10.2196/27167 (PMC8857700; doi:10.2196/27167)
Supplement: Multimedia Appendix 2 [file jmir_v24i2e27167_app2.docx]

**Multimedia Appendix 2**

**Table S2.** Sociodemographic characteristics of electronic communication users versus nonusers.

| Characteristics | | All users (N=5438) | Nonusers (n=2092) | Users (n=3337) |
| --- | --- | --- | --- | --- |
| **Age categories (n)** | | | | |
|  | Young adults (<45 years) | 1318 | 392 | 926 |
|  | Middle-aged adults (45-64 years) | 2003 | 699 | 1304 |
|  | Older adults (≥65 years) | 1957 | 916 | 1041 |
| Gender (female) | | 3071 | 1156 | 1915 |
| **Education level (n)** | | | | |
|  | Less than high school | 333 | 232 | 101 |
|  | High school graduate | 946 | 544 | 402 |
|  | Some college | 1588 | 633 | 955 |
|  | College graduate or more | 2409 | 607 | 1802 |
| Marital status (married or partnered) (n) | | 2845 | 1099 | 1318 |
| **Race/ethnicity (n)** | | | | |
|  | Non-Hispanic White | 3053 | 1050 | 2003 |
|  | Non-Hispanic Black or African American | 676 | 277 | 399 |
|  | Hispanic | 730 | 314 | 416 |
|  | Other | 389 | 129 | 260 |
| Household income (≥US $50,000) (n) | | 2871 | 786 | 2085 |
| Living alone | | 1583 | 732 | 851 |
| Residency (rural) (n) | | 599 | 291 | 308 |
| Having a regular health care provider (yes) (n) | | 3723 | 1180 | 2543 |
| Use of electronic communication (yes) (n) | | 3337 | -^a^ | - |

^a^: not available
